# Deep neural network-based structural health monitoring technique for real-time crack detection and localization using strain gauge sensors

**DOI:** 10.1038/s41598-022-24269-4

**Published:** 2022-11-23

**Authors:** Jiyoung Yoon, Junhyeong Lee, Giyoung Kim, Seunghwa Ryu, Jinhyoung Park

**Affiliations:** 1 Advanced Mechatronic R&D Group, Korea Institute of Industrial Technology, Daegu, 42994 Republic of Korea; 2 Department of Mechanical Engineering, Korea Advanced Institute of Science and Technology, Daejeon, 34141 Republic of Korea; 3 Department of Mechanical Engineering, Kyungpook National University, Daegu, 41566 Republic of Korea; 4 School of Mechatronics Engineering, Korea University of Technology and Education, Cheonan, 31253 Republic of Korea

**Keywords:** Aerospace engineering, Mechanical engineering

## Abstract

Structural health monitoring (SHM) techniques often require a large number of sensors to evaluate and monitor the structural health. In this paper, we propose a deep neural network (DNN)-based SHM method for accurate crack detection and localization in real time using a small number of strain gauge sensors and confirm its feasibility based on experimental data. The proposed method combines a DNN model with principal component analysis (PCA) to predict the strain field based on the local strains measured by strain gauge sensors located rather sparsely. We demonstrate the potential of the proposed technique via a cyclic 4-point bending test performed on a composite material specimen without cracks and seven specimens with different lengths of cracks. A dataset containing local strains measured with 12 strain gauge sensors and strain field measured with a digital image correlation (DIC) device was prepared. The strain field dataset from DIC is converted to a smaller dimension latent space with a few eigen basis via PCA, and a DNN model is trained to predict principal component values of each image with 12 strain gauge sensor measurements as input. The proposed method turns out to accurately predict the strain field for all specimens considered in the study.

## Introduction

Structural damage reduces the lifespan and reliability of engineering structures such as aircraft, buildings, and bridges, and can lead to serious fatalities and economic losses. The monitoring of structural damage is essential to improve the lifetime safety, maintainability, and reliability of structures. Therefore, structural health monitoring (SHM) techniques have been developed to monitor the extraction of damage-sensitive features such as strain, acoustic emission (AE), vibration signals, and electromechanical impedance, recorded using various sensors installed on the structure and determine the current state of structural health through statistical analysis of the extracted features^1–6^. Recently, with the development of high-performance graphics processing units and parallel computing, convolutional neural networks (CNN)-based approach using the 2D images for detecting damage have been proposed^7–10^.

AE-based SHM can quantitatively identify damage by analyzing the characteristics of elastic waves in the monitored structure. However, the detected elastic waves can be very complicated and difficult to analyze because their propagation through the structure is influenced by dispersion and geometric boundaries^11^. Alternatively, vibration-based SHM is used to monitor structural damage by analyzing vibrational characteristics such as natural frequencies, mode shapes, damping, and frequency response functions. However, it is difficult to monitor dynamic structures such as aircrafts^12^ because the influence of ambient noise and vibrations increases the difficulty in extracting the vibrational characteristics and the damaged signal can be concealed by uncertainties. By utilizing the variation of the mechanical impedance under structural damage, electromechanical impedance -based SHM can identify failures by monitoring the structure’s mechanical impedance using the electrical impedance of piezoelectric (PZT) sensors attached to the monitored structure. However, because PZT sensors are brittle, external load or impact and environmental disturbances may lead to bonding defects and sensor breakage^13^. Because the measured impedance is very sensitive to temperature and environmental noise or vibration, robust compensation methods must be developed^14^. CNN-based SHM can identify pixel-level cracks in 2D images according to the functionality of a given algorithm, allowing it to detect the presence of cracks in an inspection image or to identify the precise pixels where the crack. However, it is used to detect cracks in fixed structures such as pavement and bridge deck, not dynamic structures such as aircraft because a camera device is required to collect high-resolution images of the target structures^15–17^.

Strain is a direct indicator of stress associated with damage. Hence, strain-based SHM, as realized through strain measurements using strain gauge sensors or fiber optic sensors, has gained increasing popularity as a reliable method for real-time strain measurements. In addition, because cracks are an important feature for determining the damage condition of structures, strain-based crack monitoring techniques for crack detection provide a qualitative indication of the presence of cracks and enable their localization. These techniques are already in use for SHM approaches in the aerospace industry and have been extensively researched in the last 2 decades. Ramdane et al. theoretically and experimentally investigated the identification of crack positions, inclinations, and lengths, as well as the magnitudes of external loading based on data from 8 to 12 strain gauges distributed along the edges of a rectangular plate by using the concept of distributed dislocations in conjunction with a genetic algorithm (GA)^18,19^. Haim et al. investigated the detection of straight cracks, circular holes, and holes with arbitrary shapes based on strain measurement sensors by using the extended finite element method and genetic algorithms (GA)^20,21^. Liang et al. investigated the identification of holes and cracks in composite plates for multiple static loading modes by using strain gauge sensors and a nonlinear optimization program that applied the boundary element method and genetic algorithm^22^. Yong et al. proposed a crack detection and localization method for elastic structures that combined a rectangular strain rosette structure with three fiber Bragg gratings (FBGs), body force method, and an improved particle swarm optimization (PSO) algorithm^23^.

Thus, previous strain-based SHM studies applied optimization algorithms such as GA and PSO, using a small number of strain gauge sensors or FBG sensors to identify the crack detection and localization of structures. In this case, the crack damage cannot be monitored in real time because the optimization algorithm requires a large number of iterations to converge to the actual crack location and length. If the crack damage is not discovered and repaired in time, the service life of the structure will be reduced and the maintenance cost will increase. Therefore, real-time detection and localization of crack damage is an important requirement.

In this paper, we propose a machine learning-based method for accurately detecting and localizing cracks in real time using a small number of strain gauge sensors. The feasibility of the approach was verified based on experimental data. Because stress concentration occurs at the crack tip, it is possible to detect the cracks, determine their position and length, and monitor their growth through real-time strain field analysis. For specimens without damage and specimens with various types of damage, a dataset of local strains measured with 12 strain gauge sensors and their corresponding strain field maps over a wide domain measured with digital image correlation (DIC) devices were used to train and evaluate the DNN model performance. The high dimensional strain field map (14 $\times$ 12 pixel image) is compressed into a smaller dimension latent space via PCA to reduce the output dimension for the DNN . The trained DNN takes the 12 strain gauge measurements as input and accurately predict the strain field map over a wide domain. The feasibility of the proposed method is demonstrated for real-time SHM for cyclic 4-point bending test.

**Figure 1:**
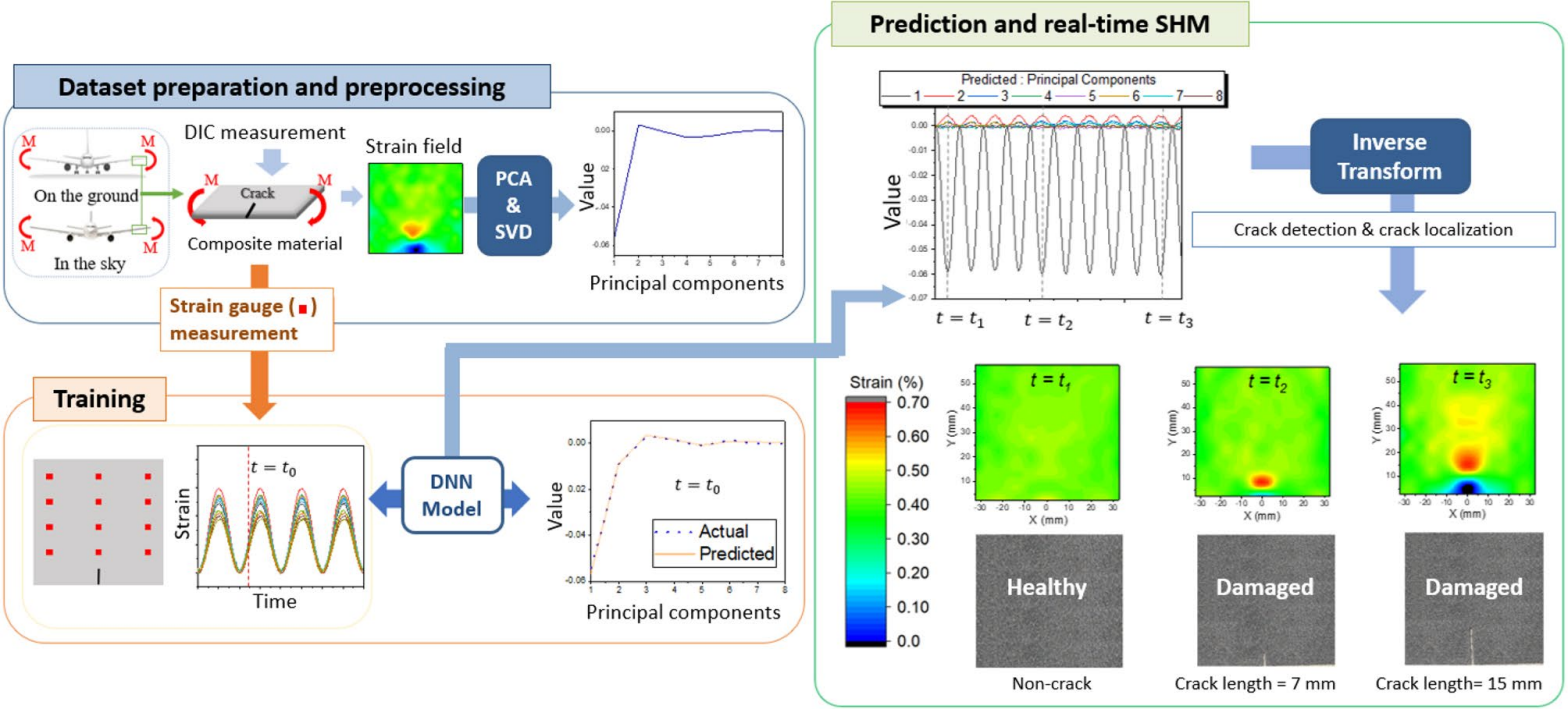
Framework of the proposed method for real-time structural health monitoring using strain gauge sensors.

## Methodology

Figure 1 shows the real-time crack-damage monitoring system, which predicts the strain field through a machine learning model with a small number of strain gauge sensors. The target monitoring structure is an aircraft.When the aircraft is on the ground, resting on its landing gear, the force of gravity attempts to bend the wing downward. An aircraft in flight experiences a bending force on its wing as an aerodynamic lift attempts to raise the wing^24^. Therefore, the aircraft wing is subjected to cyclic bending moment. We prepared a dataset consisting of local strains measured with 12 strain gauges and strain field measured with the DIC device for one specimen without damage (Healthy) and seven specimens with various lengths of edge cracks (Damaged) under a cyclic bending moment. The specimen was made of a composite material laminated carbon fiber-reinforced plastic (CFRP) generally used in aircraft^25^. To preprocess the strain field, we represented all the strain field map from DIC in 168 dimensions (14 $\times$ 12 pixel image) and reduced them to eight dimensions (referred to as eight principal component values, hereafter) by employing PCA^26,27^. The dimensionality reduction process is important for reducing the computational time and enhancing the training accuracy of DNN. Finally, the DNN model used the local strains measured with the 12 strain gauge sensors as input and predicted the eight principal component values. The DNN model prediction was transformed back into a regular strain field. By analyzing the predicted strain field, the feasibility of real-time monitoring of crack detection and localization was verified.

**Figure 2:**
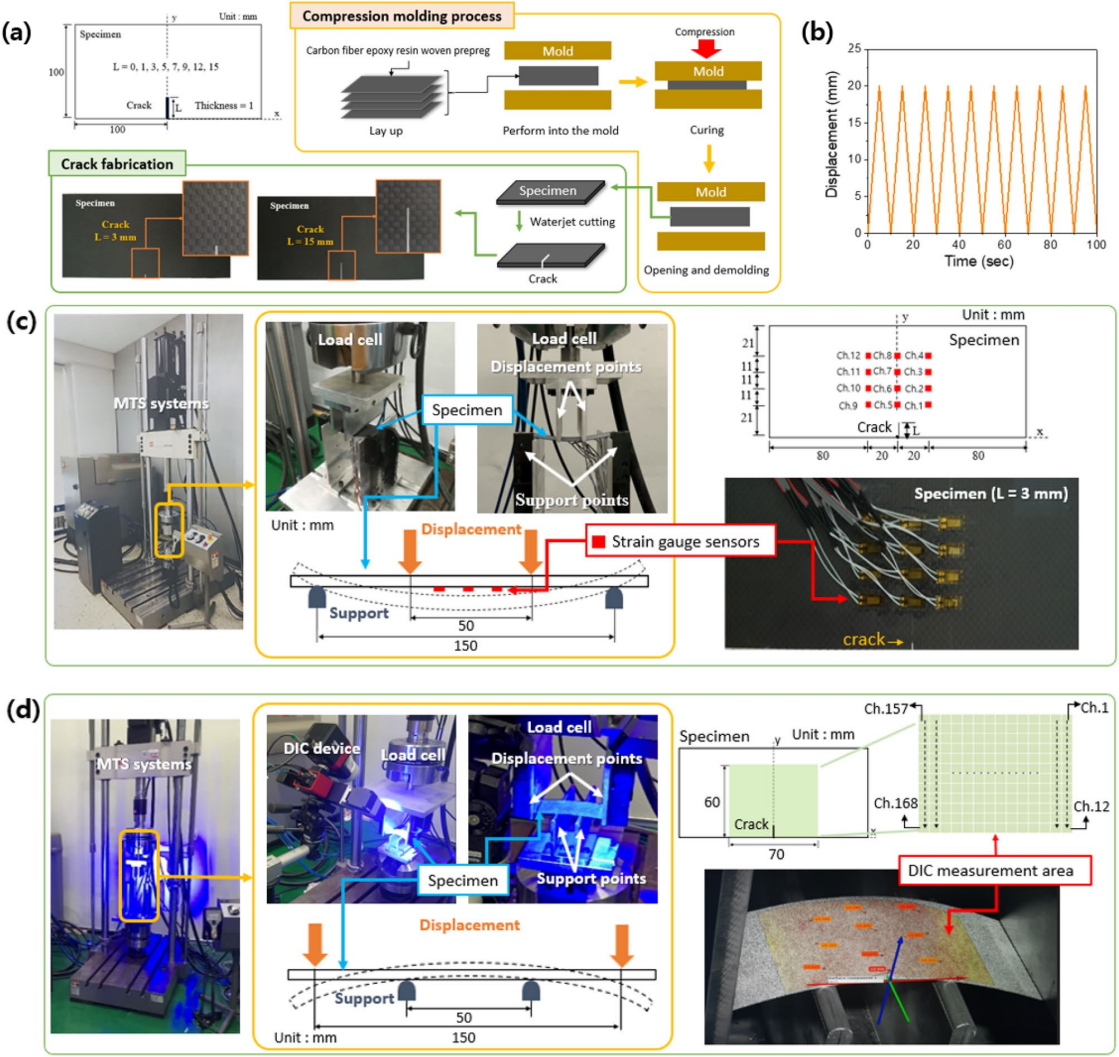
Experimental setup (**a**) Schematic of the specimen geometry and preparing process of the specimens, (**b**) Cyclic 4-point bending, (**c**) strain measured with strain gauge sensors, (**d**) strain field measured with DIC device.

### Experiments

This section presents the experimental process for preparing the training, validation, and testing datasets for crack detection and localization of the composite using machine learning.

#### Specimen preparation

Figure 2a shows the schematic of the specimen geometry and preparing process of the specimens. The dimensions of the specimens were 200 mm (length) × 100 mm (width) × 1 mm (height). Eight specimens were prepared, one crack-free healthy specimen and seven damaged specimens with cracks of different lengths at a fixed position. The CFRP specimen was produced by compression molding a 4-ply carbon fiber epoxy resin woven prepreg with a thickness of 0.26 mm. In the curing cycle for this prepreg, the temperature was ramped up to 125 °C and held for 2 h before allowing the material to cool in a conventional thermal heating environment. The in-plane Young’s modulus of the composite material was 60 GPa. All specimens with cracks were prepared using a waterjet cutting machine for crack fabrication. The crack was located at the edge of the symmetry line in the rectangular specimen.

#### Experimental setup

A cyclic 4-point bending test was performed using a uniaxial hydraulic testing machine (MTS Systems) to determine the strain of the specimens. Loading was applied for 10 cycles with displacement control from 0 to 20 mm at a speed of 0.1 Hz (Fig. 2b). Figure 2c depicts the experimental setup for measuring the strain in the x-direction using uniaxial strain gauge sensors (KFGS series, Kyowa Electronics). The strain gauge sensors consist of insulating flexible backing that supports a metallic foil pattern. When a specimen with a strain gauge sensor is deformed, the metallic foil is deformed, causing its electrical resistance to change. The strain is derived from the resistance changes measured using a Wheatstone bridge. The experimental setup for measuring the strain field in the x-direction using a DIC device (GOM Aramis) is presented in Fig. 2d^28^. The DIC technique measures the displacement and deformation of a specimen by using a digital camera. It involves forming a random speckle pattern on the surface of a specimen, and comparing the images before and after the deformation to derive the displacement, deformation, and strain. Pixel information with 256 Gray levels was formed on the measured area of the specimen. A speckle pattern should be formed on the surface of an object before deformation because the larger the contrast, the more accurate is the information contained in a unit pixel. When the object is deformed, this pattern is deformed and the amount of deformation is measured in pixels. A group of pixels forms a subset and acts as a hypothetical strain gauge. The DIC technique can be considered as the continuous measurement of a subset in a continuous image.

The experimental setup consisted of two upper displacement points moving in the vertical direction, and two lower support points fixed on the table. Since the behavior of the specimen could not be simultaneously measured using the strain gauge sensors and the DIC device, the positions of the upper displacement points and lower support points were different in the experiments to measure the same deformation behavior in both cases. Twelve strain gauge sensors were used, and 168 subsets were applied to the strain field measured using the DIC device. The training, validation, and test datasets were created with a sampling rate of 5 Hz. Considering the error of the experiment, a cyclic 4-point bending test was performed with five specimens for each specimen type (healthy and damaged), and a dataset of 20,000 data was obtained.

### Deep neural network (DNN) model

This section presents the machine learning process to predict the strain field in real time using strain gauges based on the datasets obtained in the previous section.

#### Modelling the strain filed by DIC with PCA

Because of the high dimensionality of strain field data with 168 subsets (DIC generates 14 × 12 pixel image of strain field) , PCA is employed to reduce the dimensionality of the strain field. PCA reduces the dimensions of a dataset consisting of a large number of interrelated variables while maintaining the variation present in the dataset to the extent possible. This is achieved by converting a new set of variables into ordered principal components such that the first few are uncorrelated and retain most of the variations present in all original variables.

Out of 10,000 DIC images with 168 dimensions, we can construct a 10,000 × 168 strain matrix (denoted as the *X* matrix), where each row represents a strain field at a given time and each column represents the strain value according to time at a given subset. PCA is performed by employing the singular value decomposition (SVD) shown in Eq. (1); it is a real matrix factorization method similar to the reliable and robust orthogonal matrix decomposition technique. Here, *U* and *V* are left and right singular vectors, respectively. All off-diagonal elements of the $\Sigma$ matrix are zero, and the diagonal elements are known as singular values $\sigma _i$ of Eq. (2). The $\Sigma ^T\Sigma$ matrix contains the eigenvalues $\lambda _i$ of Eq. (3), which determines the variance explained for each principal component. The $V^T$ matrix contains the principal components in the order in which the variance decreases. The first n principal components (the singular value n) were retained and the rest were discarded. This is achieved by projecting the data along the singular value of $V^T$ to obtain an n-dimensional representation of the strain field. The singular value should be selected to approximately describe the *X* matrix. This can be determined by examining the cumulative explained variance ratio as a function of the singular value defined in Eq. (4)^29,30^.

1 $$\begin{aligned}{} & {} X=U\Sigma V^T \end{aligned}$$

2 $$\begin{aligned}{} & {} \Sigma =diag(\sigma _1,\sigma _2,\ldots ,\sigma _{168})\end{aligned}$$

3 $$\begin{aligned}{} & {} (\sigma _i)^2=\lambda _i \end{aligned}$$

4 $$\begin{aligned}{} & {} C.E.V.(n)=\frac{\sum _{i=1}^n\lambda _i}{\sum _{j=1}^{168}\lambda _j} \end{aligned}$$

**Figure 3:**
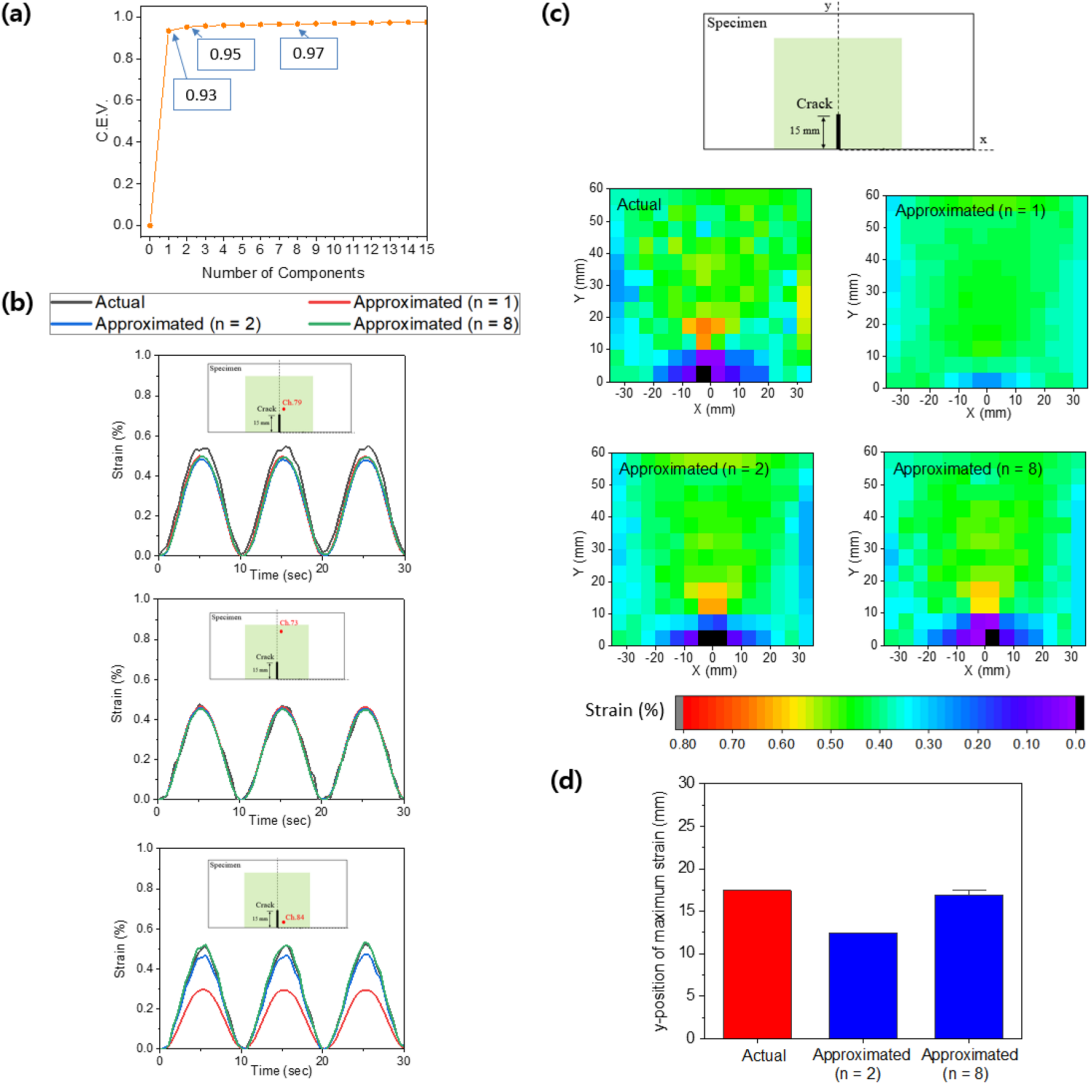
(**a**) Screen plot of PCA, i.e., cumulative explained variance ratio according to the number of components, (**b**) comparison of actual strain and approximated strain, (**c**) comparison of actual strain field and approximated strain field, (**d**) Y-position of maximum strain.

**Figure 4:**
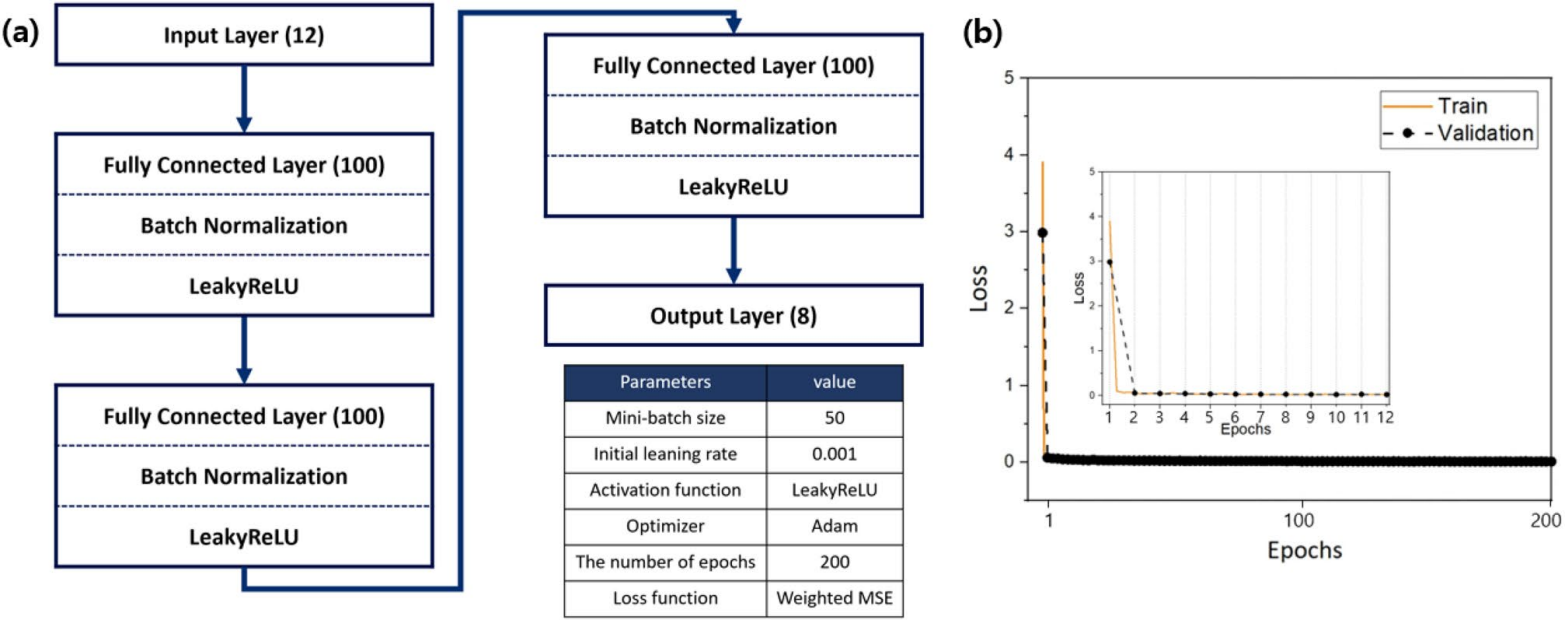
(**a**) Network structure and parameters of the DNN model, (**b**) curve of loss function during DNN model training.

In Fig. 3a, the usage of singular values up to $\sigma _1$, $\sigma _2$, and $\sigma _8$ (n = 1, 2, and 8) have cumulative explained variances of 93%, 95%, and 97%, respectively. Figure 3b shows the strain measured at subsets 73, 79, and 84 in the specimen with an edge crack length of 15 mm for the three singular values. Although the difference in the cumulative explained variance ratio was not large among them, significant error in strain estimation occurs at the pixels closer the closer the crack. Figure 3c shows the actual strain field from DIC and approximated strain field in the specimen with a crack length of 15 mm when the displacement load was 20 mm, which clearly shows that the improvement of strain field estimation with more singular values. Because stress concentration occurred at the crack tip, the position of maximum strain occurrence was considered as the crack tip. For precise crack localization, the position of maximum strain in the approximated strain field should correspond to the actual point of maximum strain. Figure 3d shows a comparison of the actual points of maximum strain for five specimens with a crack length of 15 mm and the approximated point of maximum strain for n = 2 and n = 8. The singular value was selected as 8 because the position of occurrence of maximum strain and strain distribution in the approximated strain field were close to the actual values. Thus, we converted the 168-dimensional strain field image from the DIC into 8-principal component values which allows us to more efficient train a DNN model.

#### DNN model design and training

The DNN model was trained to predict eight principal component values with local strains measured at 12 strain gauge sensors as inputs. The input and output features of the DNN model were normalized using min-max normalization. The DNN model used in this study comprised an input layer, three hidden layers, and an output layer (Fig. 4a). The neurons in two adjacent layers were fully connected. Each connection was assigned a trainable weight multiplied by the input value. All hidden layers used the Leaky ReLU activation function, followed by batch normalization. The weight and bias values (parameters) were initialized using Xavier initialization. The mini-batch size was set to 50, and the number of epochs to 200. The Adam optimizer was used to update the DNN parameters during backpropagation, and the initial learning rate was set to 0.001^31,32^. Additionally, the learning rate was dropped 10 times every 50 epochs for fine tuning of the neural network hidden layer. The loss function plays an important role in the construction of the DNN model and improvement of its performance because it is an indicator of the difference between the actual and predicted values. We selected the weighted mean squared error loss functions based on the importance weighting in Eq. (4)^33^:

5 $$\begin{aligned}{} & {} Weighted\,MSE=\sum _{i=1}^n\sum _{j=1}^8 \frac{\lambda _j}{\sum _{k=1}^{168}\lambda _k}(y_i[j]-\widehat{y_i[j]})^2 \end{aligned}$$

In other words, the weighted mean squared error loss function trains the DNN model such that certain components are more important than the rest by using the ratio of the eigenvalues that describe the explained variance of each component. The experimental results of the three specimens for each specimen type were used as the training/validation datasets (12,000), and those of the remaining two specimens as the test datasets (8000). The splitting ratio for the training/validation dataset was set at 9:1. Figure 4b shows the convergence of loss function over training and validation data.

**Figure 5:**
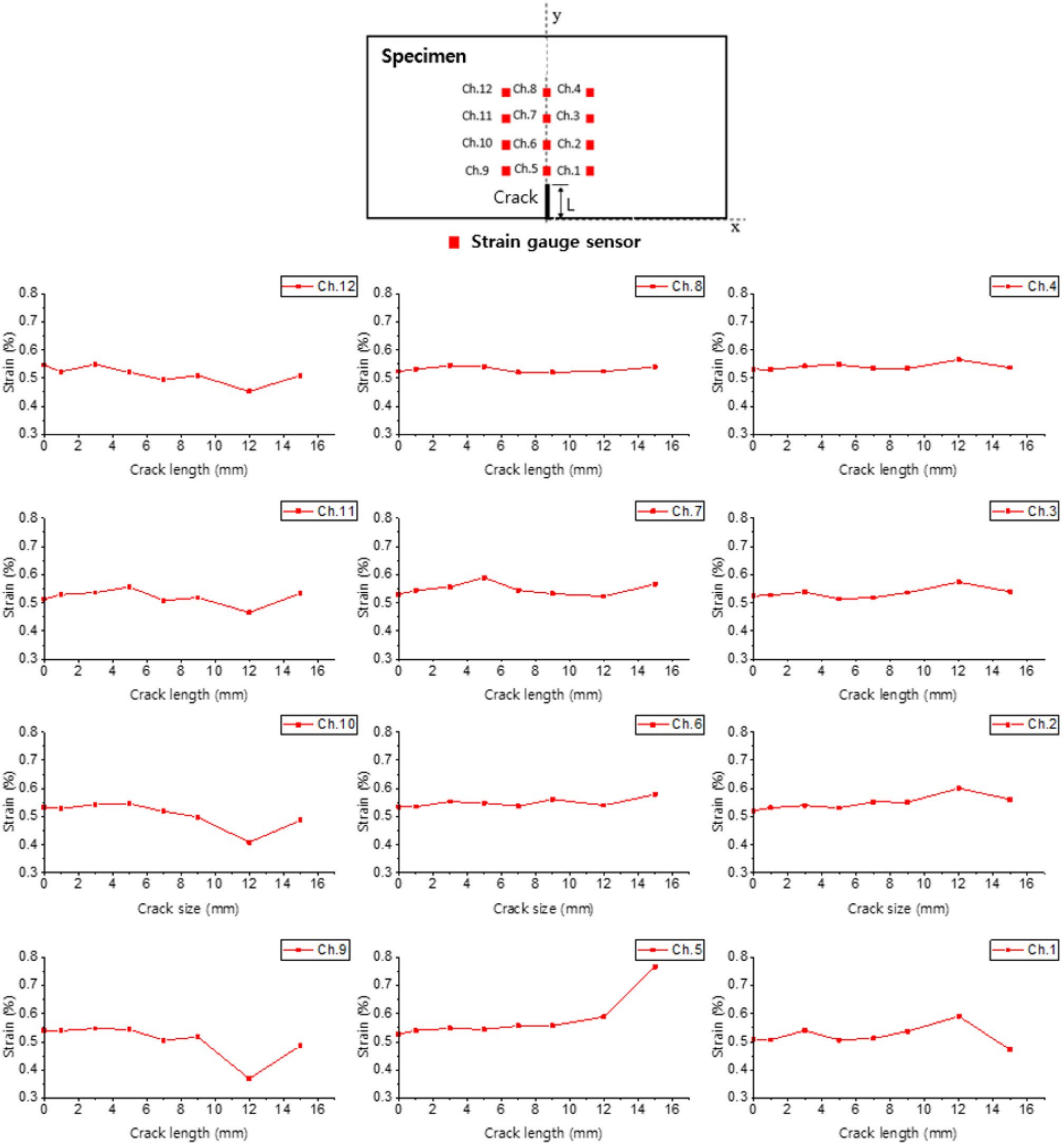
Strain measured with strain gauge sensors according to crack length.

## Results and discussion

This section presents the performance of the proposed method for crack detection and localization.

### Comparison of measurement results of strain gauge sensors and DIC device

Figure 5 shows the local strain values measured from 12 strain gauge sensors at the maximum displacement load during the cyclic loading tests for specimens with different crack lengths. The strain value (y-axis) corresponds to the averages of the top 50 values for specimens with given crack length. The x-axis represents the crack length, and can be regarded as the time axis through which the crack propagates if we presume to perform a fatigue test. The strain in channel 5 was maintained at 0.5% when the crack length was 12 mm or less, and it was measured to be 0.8% when the crack length was 15 mm. A sudden strain change in channel 5 of the strain gauge indicated that a crack was detected nearby.

The strain of all channels, except channel 5, did not increase rapidly with the crack length. Because the stress was concentrated at the crack tip, a sudden increase in the strain according to Hook’s law indicated that cracks were detected. Considering the crack length and position of channel 5, it can be seen that the strain gauge sensor can only detect cracks existing in an area within 7 mm, and it is difficult to accurately estimate the damage localization such as crack position and crack length. It is possible to use a large number of strain gauge sensors to estimate the crack localization, but this is impractical, as it requires significant effort and cost.

**Figure 6:**
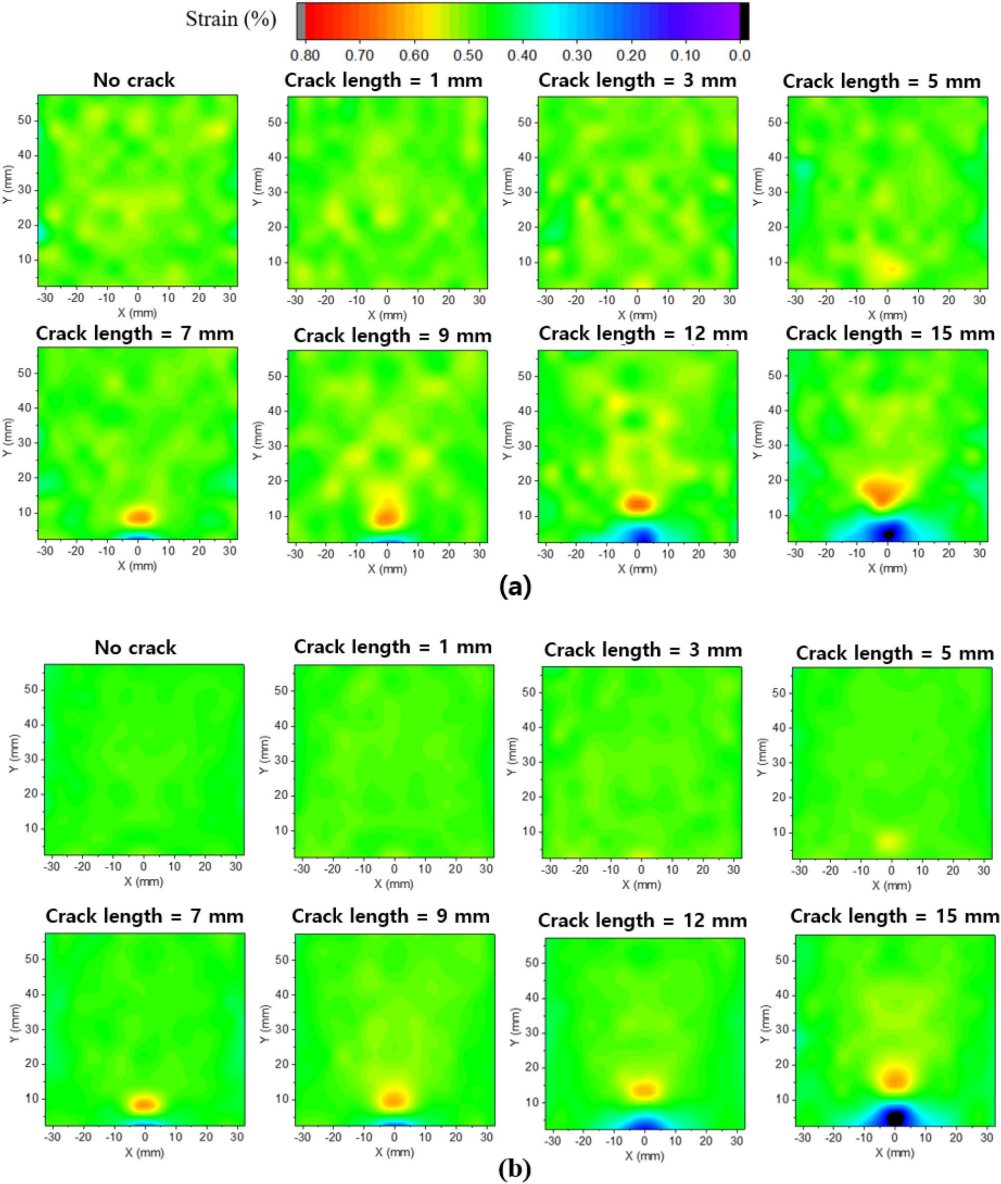
Comparison of actual and predicted strain fields (**a**) actual strain field measured with DIC device, (**b**) strain field predicted by the DNN model.

Figure 6a shows the strain field results measured using the DIC device for each crack length under the maximum displacement load. Because we used 168 subsets for the DIC measurement owing to the data storage problem, the subset size was 5 mm (length) $\times$ 5 mm (width). Therefore, the strain field was represented using Delaunay triangulation^34^ with linear interpolation to accurately perform the crack detection and localization. A comparison of the strain fields with and without cracks shows that the strain concentration appears at the crack length of 3 mm, and it occurs near the crack tip based on which the crack length can be estimated. Thus, it can be seen that crack detection and localization are possible with the current DIC subset size for crack lengths of 3 mm or more.

### Damage detection and localization

To examine the performance of the trained and validated DNN model, we used 8000 test dataset that were not utilized in the training and validation processes. The DNN model prediction in the 8-dimensional latent space was transformed back into a strain field. The predicted strain fields were then compared with the actual strain field measured using a DIC device. The regression evaluation metrics of R-squared value ($R^2$), mean absolute error (MAE), and root mean squared error (RMSE) were used as the DNN model performance metrics^35^. In addition, because the crack detection, position, and length can be predicted based on the position of maximum strain, the prediction accuracy for the position of maximum strain was also adopted as a DNN model performance metric. The performance results of the DNN model are listed in Tables 1 and 2. In Table 1, $R^2$, also known as the coefficient of determination, is an indicator of the extent to which an independent variable (predicted value) explains the dependent variable (actual value) in a regression model. In this study, $R^2$ was 0.96, which means that the strain field predicted by the DNN model could explain 96% of the strain field measured using the DIC device. MAE assigns the same weight to the error in all cases by calculating the average of the absolute values of the difference between the predicted and actual values. RMSE is calculated as the square root of the average of the squares of all errors; thus, each error has a different weight. Because the MAE is an intuitive indicator and the RMSE is an outlier-sensitive indicator, we examined these two types of errors. Considering the typical strain gauge measurement error of 5% or less and the strain range measured in this study, the MAE and RMSE were within the measurement error range. Additionally, the predicted position of maximum strain for each crack length matched the actual position by more than 90% from Table 2. Thus, the DNN model could effectively predict the strain field measured with the DIC device.

Figure 6b shows the strain field predicted by the DNN model for each crack length under the maximum displacement load. The strain field predicted by the DNN model was similar to that measured by the DIC device. The strain field predicted from the DNN model is smoother than the original image from the DIC device because the measurement noise was eliminated through PCA dimension reduction, which is beneficial in damage detection and localization.

**Table 1:** DNN model performance metrics.

Metrics	Value
R-squared ($R^2$)	0.96
RMSE	0.029
MAE	0.021

**Table 2:** Accuracy of predicted position of maximum strain for each crack length.

Crack length (mm)	Accuracy ($\%$)
1	96.6
3	95.5
5	91.8
7	92.5
9	96.3
12	97.3
15	95.4

## Conclusion

We proposed a real-time crack monitoring method for a composite material by combining DNN with PCA and SVD and predicting the strain field based on local strains measured using 12 strain gauge sensors. The feasibility of the proposed method was demonstrated based on experimental data following the length of the edge crack under the restriction of fixed crack position. When only 12 strain gauge sensors were used, crack detection was impossible when the length of edge crack was 12 mm or less. However, when our proposed method was applied, crack length of at least 3 mm could be detected, and the exact location and length of the detected cracks were also accurately monitored. The proposed method can be extended to the application of various crack parameters such as crack position, length, and inclination in the future. To accurately identify the crack detection and localization in real time, a large number of strain gauge sensors are required, which necessitate a lot of effort and cost; so it is difficult to apply. However, our proposed DNN based-SHM technique will contribute to strain-based SHM of engineering structures such as aircrafts because it is possible to accurately identify the crack detection and localization in real time through a small number of strain gauge sensors.

## Data Availability

The datasets used and/or analyzed during the current study available from the corresponding author on reasonable request.

## References

[1] Farrar CR, Worden K (2007). An introduction to structural health monitoring. Philos. Trans. R. Soc. A Math. Phys. Eng. Sci..

[2] Abdulkarem M, Samsudin K, Rokhani FZ, Rasid MF (2020). Wireless sensor network for structural health monitoring: A contemporary review of technologies, challenges, and future direction. Struct. Health Monit..

[3] Güemes A, Fernández-López A, Díaz-Maroto PF, Lozano A, Sierra-Perez J (2018). Structural health monitoring in composite structures by fiber-optic sensors. Sensors.

[4] Seguel F, Meruane V (2018). Damage assessment in a sandwich panel based on full-field vibration measurements. J. Sound Vib..

[5] Gulizzi V, Rizzo P, Milazzo A, LaMalfaRibolla E (2015). An integrated structural health monitoring system based on electromechanical impedance and guided ultrasonic waves. J. Civ. Struct. Health Monit..

[6] Bhuiyan MY, Bao J, Poddar B, Giurgiutiu V (2018). Toward identifying crack-length-related resonances in acoustic emission waveforms for structural health monitoring applications. Struct. Health Monit..

[7] Dorafshan S, Thomas RJ, Maguire M (2018). Comparison of deep convolutional neural networks and edge detectors for image-based crack detection in concrete. Constr. Build. Mater..

[8] Kim B, Yuvaraj N, Sri Preethaa K, Arun Pandian R (2021). Surface crack detection using deep learning with shallow CNN architecture for enhanced computation. Neural Comput. Appl..

[9] Quqa S (2022). Two-step approach for fatigue crack detection in steel bridges using convolutional neural networks. J. Civ. Struct. Health Monit..

[10] Bai Y, Zha B, Sezen H, Yilmaz A (2022). Engineering deep learning methods on automatic detection of damage in infrastructure due to extreme events. Struct. Health Monit..

[11] Kral Z, Horn W, Steck J (2013). Crack propagation analysis using acoustic emission sensors for structural health monitoring systems. Sci. World J..

[12] Yang Y, Zhang Y, Tan X (2021). Review on vibration-based structural health monitoring techniques and technical codes. Symmetry.

[13] Ai D, Luo H, Zhu H (2017). Diagnosis and validation of damaged piezoelectric sensor in electromechanical impedance technique. J. Intell. Mater. Syst. Struct..

[14] Tenreiro AFG, Lopes AM, da Silva LF (2022). A review of structural health monitoring of bonded structures using electromechanical impedance spectroscopy. Struct. Health Monit..

[15] Zhang Q, Barri K, Babanajad SK, Alavi AH (2020). Real-time detection of cracks on concrete bridge decks using deep learning in the frequency domain. Engineering.

[16] Ren Y (2020). Image-based concrete crack detection in tunnels using deep fully convolutional networks. Constr. Build. Mater..

[17] Fan, R. *et al.* Road crack detection using deep convolutional neural network and adaptive thresholding. In *2019 IEEE Intelligent Vehicles Symposium (IV)*, 474–479 (IEEE, 2019).

[18] Boukellif R, Ricoeur A (2020). Identification of crack parameters and stress intensity factors in finite and semi-infinite plates solving inverse problems of linear elasticity. Acta Mech..

[19] Boukellif R, Ricoeur A, Oxe M (2021). Parameter identification of crack-like notches in aluminum plates based on strain gauge data. Struct. Health Monit..

[20] Waisman H, Chatzi E, Smyth AW (2010). Detection and quantification of flaws in structures by the extended finite element method and genetic algorithms. Int. J. Numer. Methods Eng..

[21] Rabinovich D, Givoli D, Vigdergauz S (2009). Crack identification by ‘arrival time’ using XFEM and a genetic algorithm. Int. J. Numer. Methods Eng..

[22] Liang Y-C, Sun Y-P (2020). Hardware-in-the-loop simulations of hole/crack identification in a composite plate. Materials.

[23] Chen Y, Liu Z-Q, Liu H-L (2018). Parameters identification for crack in elastic structures based on fiber bragg grating. Optik.

[24] Ma Z, Chen X (2018). Fiber bragg gratings sensors for aircraft wing shape measurement: Recent applications and technical analysis. Sensors.

[25] Andersson F, Hagqvist A, Sundin E, Björkman M (2014). Design for manufacturing of composite structures for commercial aircraft-the development of a DFM strategy at SAAB aerostructures. Proced. Cirp.

[26] Jolliffe IT, Cadima J (2016). Principal component analysis: A review and recent developments. Philos. Trans. R. Soc. A Math. Phys. Eng. Sci..

[27] Kuang L (2014). A tensor-based approach for big data representation and dimensionality reduction. IEEE Trans. Emerg. Top. Comput..

[28] Yoneyama S (2016). Basic principle of digital image correlation for in-plane displacement and strain measurement. Adv. Compos. Mater.

[29] Gerbrands JJ (1981). On the relationships between SVD, KLT and PCA. Pattern Recogn..

[30] Berrar DP, Dubitzky W, Granzow M (2003). A Practical Approach to Microarray Data Analysis.

[31] Montavon G, Samek W, Müller K-R (2018). Methods for interpreting and understanding deep neural networks. Digital Signal Process..

[32] Azimi M, Eslamlou AD, Pekcan G (2020). Data-driven structural health monitoring and damage detection through deep learning: State-of-the-art review. Sensors.

[33] Yang C, Kim Y, Ryu S, Gu GX (2020). Prediction of composite microstructure stress-strain curves using convolutional neural networks. Mater. Design.

[34] Renka RJ (1984). Interpolation of data on the surface of a sphere. ACM Trans. Math. Softw..

[35] Chicco D, Warrens MJ, Jurman G (2021). The coefficient of determination r-squared is more informative than SMAPE, MAE, MAPE, MSE AND RMSE in regression analysis evaluation. PeerJ Comput. Sci..

